# Nitrates in drinking water and methemoglobin levels in pregnancy: a longitudinal study

**DOI:** 10.1186/1476-069X-9-60

**Published:** 2010-10-14

**Authors:** Deana M Manassaram, Lorraine C Backer, Rita Messing, Lora E Fleming, Barbara Luke, Carolyn P Monteilh

**Affiliations:** 1 Centers for Disease Control and Prevention, National Center for Environmental Health, Health Studies Branch. 4770 Buford Highway, MS F-57 Chamblee, GA 30341, USA; 2 University of Miami, Department of Epidemiology and Public Health, 1120 NW 14th Street Miami, FL 33136, USA; 3 Minnesota Department of Health, Division of Environmental Health, 625 N. Robert St. St. Paul, MN 55155, USA

## Abstract

**Background:**

Private water systems are more likely to have nitrate levels above the maximum contaminant level (MCL). Pregnant women are considered vulnerable to the effects of exposure to high levels of nitrates in drinking water due to their altered physiological states. The level of methemoglobin in the blood is the biomarker often used in research for assessing exposure to nitrates. The objective of this study was to assess methemoglobin levels and examine how various factors affected methemoglobin levels during pregnancy. We also examined whether differences in water use practices existed among pregnant women based on household drinking water source of private vs. public supply.

**Methods:**

A longitudinal study of 357 pregnant women was conducted. Longitudinal regression models were used to examine changes and predictors of the change in methemoglobin levels over the period of gestation.

**Results:**

Pregnant women showed a decrease in methemoglobin levels with increasing gestation although <1% had levels above the physiologic normal of 2% methemoglobin, regardless of the source of their drinking water. The multivariable analyses did not show a statistically significant association between methemoglobin levels and the estimated nitrate intake from tap water among pregnant women around 36 weeks gestation (β = 0.046, p = 0.986). Four women had tap water nitrate levels above the MCL of 10 mg/L. At enrollment, a greater proportion of women who reported using water treatment devices were private wells users (66%) compared to public system users (46%) (p < 0.0001). Also, a greater proportion of private well users (27%) compared to public system users (13%) were using devices capable of removing nitrate from water (p < 0.0001).

**Conclusion:**

Pregnant women potentially exposed to nitrate levels primarily below the MCL for drinking water were unlikely to show methemoglobin levels above the physiologic normal. Water use practices such as the use of treatment devices to remove nitrates varied according to water source and should be considered in the assessment of exposure to nitrates in future studies.

## Background

While there have been improvements in the quality and safety of drinking water, in part due to the U.S. Safe Water Drinking Act (SWDA), adverse health effects from drinking water contaminants continue to pose a significant problem [1-8]. The U.S. Environmental Protection Agency (U.S. EPA) set the maximum contaminant level (MCL) for nitrates in public drinking water at 10-mg/L nitrate-nitrogen (NO_3_-N) to protect infants from methemoglobinemia. However, this MCL does not incorporate a built-in safety factor as it is based on methemoglobin formation in infants exposed to water containing greater than10 mg/L nitrate [9]. There is a lack of data on whether exposure to nitrate below the MCL will produce adverse effects where no clinical symptoms have been detected, particularly with chronic exposure. Furthermore, private drinking water systems are not regulated and may be more vulnerable to nitrate contamination, particularly in areas of intense agricultural activities [10,11].

Methemoglobinemia is an anemia resulting from the oxidation of the ferrous iron in hemoglobin to the ferric state, changing hemoglobin to methemoglobin [12]. Methemoglobin cannot carry molecular oxygen therefore if it is produced at a higher rate than the body is able to convert back to hemoglobin, this can lead to cyanosis, tissue hypoxemia and in severe cases, death. The normal physiological concentration of methemoglobin in the blood is less than 2% of total hemoglobin, however symptoms of methemoglobinemia may not be apparent until levels are at or above 10% [13]. Acquired methemoglobinemia can result from exposure to certain pharmaceutical preparations (e.g. lidocaine, benzocaine, sulfonamides, dapsone, nitroglycerine) or chemical substances (e.g. nitrates, copper, sulfate, chlorite, chloramines and chlorates), which may cause oxidation of hemoglobin to methemoglobin faster than methemoglobin is reduced back to hemoglobin [14].

Acquired methemoglobinemia due to exposure to nitrates in drinking water is considered primarily an issue for infants less than six months old [15]. Pregnant women are also considered vulnerable to the effects from exposure to high levels of nitrate in drinking water [16,17], and reports suggest an association between environmental nitrate exposure with both adverse effects during pregnancy and adverse birth outcomes [18-20]. There is no information on the effects of chronic exposure to low doses of nitrates (below the MCL) in drinking water on methemoglobin levels in infants and pregnant women. Subclinical increases in methemoglobin levels may occur in populations exposed to low levels of nitrates in drinking water; however, methemoglobin levels in these populations have not been well characterized. Furthermore, little is known about methemoglobin levels during pregnancy and whether intake of nitrates impacts blood methemoglobin levels in pregnant women.

Although the literature on the effects of nitrates on methemoglobinemia and maternal health is sparse, reports are suggestive of an association between nitrates in drinking water and spontaneous abortion [21,22], pregnancy complications [18], intrauterine growth restriction and prematurity [23]. It has been observed that the oxidation of hemoglobin to methemoglobin by nitrogen compounds such as nitrates, can impair the overall oxygen carrying capacity of the blood resulting in the oxidizing of tissue components and suppression of the antioxidant defense system [24,25]. The simultaneous exposure to environmental hazards such as nitrates during pregnancy could escalate oxidative stress and deplete antioxidant reserves, thus increasing the risk of adverse prenatal effects [26]. Therefore, pregnant women may be more sensitive to the induction of clinical methemoglobinemia by exposure to nitrates levels below the MCL [17].

We conducted a prospective investigation that incorporated individual assessment of exposure to evaluate changes in methemoglobin levels in women during pregnancy, and how various factors affected those levels, including maternal exposure to nitrates in drinking water. We also examined water use practices between pregnant women on different household drinking water sources.

## Methods

### Study Design and Population

This study was a longitudinal study of pregnant women who were recruited while receiving prenatal care at a Community Hospital health clinic in south-central Minnesota between May 2004 and September 2005. The clinic study site served women from seven primarily rural neighboring counties with small towns. While the use of private wells is substantial in the study area, water for domestic use is also provided by municipal water systems or regulated community wells. The municipal water systems and community wells in the area are subject to U.S.EPA regulations.

### Approvals

This study protocol was approved by the Institutional Review Boards of the Centers for Disease Control and Prevention (CDC) and the Minnesota Department of Health (MDH).

### Selection and Recruitment

All pregnant women who were at least 18 years of age, in their first trimester of pregnancy (≤13 weeks gestation), and seeking prenatal care at the participating clinic were eligible to participate. Pregnant women who agreed to participate provided written informed consent. Enrollment was sequential with one woman using a public water system enrolled for every two women with private well serving one home. A public drinking water system was defined as a system serving at least 25 people or has 15 service connections and was regulated under the SDWA [27]. Water source was defined as the primary source of the water supplied to the home for household use.

### Interviews

Women were evaluated at enrollment, around 20, 28, 36 weeks gestation, the day of delivery, and 2-4 weeks postpartum. With the exception of the day of delivery interview, time periods (within ± 14 days) around the gestational age of interest were used in an effort to coordinate with the participants' prenatal clinic visits, minimize missing data, and minimize loss-to-follow-up. All interviews were administered using a computerized assisted in-person interviewing (CAPI) system. The 36-week and postpartum interviews were done at the participant's home to facilitate water sample collection and confirm water source and type of treatment device. Information about residence, primary drinking water source, water treatment devices, drinking water use patterns, smoking, use of medications that could affect methemoglobin levels such as nitrosatable drugs or anesthetics, and gastrointestinal illness within the past 24 hours was collected at each follow up. At each interview information was also obtained on whether a selected list of foods with potentially high nitrate levels were eaten in the past 24 hours (specifically, cured meats, spinach, carrots, beets, potatoes, celery, cabbage, lettuce, turnips, broccoli, radishes, callard or mustard greens). At the 36 weeks gestation visit, we obtained socio-demographic information, employment history, and occupational exposures.

### Methemoglobin Testing

Blood methemoglobin levels were measured via finger stick at each visit. The testing was performed immediately after collection using the portable *AVOXimeter 4000 *(Avox Systems, Inc., San Antonio, TX) whole blood oximeter device, which measures the total hemoglobin and the percentage that is in the form of oxyhemoglobin, carboxyhemoglobin, or methemoglobin.

The manufacturer reported an accuracy of ± 0.5% and precision of ± 0.7%. Over the study period, four instruments were used for methemoglobin testing at the clinic sites and home visits. To verify the accuracy of the AVOXimeter 4000 co-oximeter, each month two participants having their blood tested on an instrument were asked to give a venous blood sample for methemoglobin testing at the local Community Hospital Lab using their standard methods. The results obtained from the Hospital labs were compared to the co-oximeter results for QA/QC to verify the margin of error as recommended by the manufacturer. Calibration of the instruments were performed at the beginning of each day, and quality control testing was performed before testing each sample using optical standards and controls provided by the manufacturer. Methemoglobin was reported as a percentage of total hemoglobin with up to 2% considered physiologic normal [12].

### Water Testing

We attempted water collection at the 36 weeks gestation and during the postpartum home visit. For some participants only one water sample was collected. In study subjects with 2 samples, the 36-week sample was used in the analyses. In cases of fetal loss (miscarriage) or delivery prior to 36 weeks gestation, efforts were made to collect and test water samples at that time.

Water samples were collected from the kitchen tap by opening the tap fully and letting the water run for 3 to 5 minutes, then reducing the water flow and filling the sample bottles. If the home had a point of entry water treatment system, or the kitchen tap had a point of use water treatment system, the sample was collected with the system in place. Cold water was collected in separate sterile bottles supplied by an independent analytical laboratory. Sample bottles were labeled with the date, time of collection, participant's study identification number, the name of the study coordinator who collected the sample, and then kept at 4°C until analyzed. Samples were tested for the presence of fecal coliform using the Colilert^® ^Presence/Absence Comparator within 24 hours of collection. All other substances were tested within 10 days of sample collection using SDWA approved methodology. Nitrate testing was done using U.S. EPA method 353.2, QuikChem system flow injection analysis. Copper was tested using Inductively Coupled Plasma/Mass Spectrometry (ICP/MS). Sulfate samples were assayed by spectrophotometry after addition of methyl thymol blue reagent.

Sulfates and copper were tested as they are possible oxidizing agents which could potentially convert hemoglobin to methemoglobin [28-30]. Bacteria in the form of total and fecal coliform were tested as previous reports showed evidence that endogenous production of nitric oxide during enteric infections resulted in the formation of methemoglobin [31-33]. If the nitrate levels in a sample were above the MCL of 10 mg/L NO_3_-N, the remaining sample was sent to the MDH, Environmental Health Laboratory, St Paul, MN, to test for pesticides. However, no detectable levels of pesticides were found in the samples tested.

### Statistical Analyses

Data processing and analyses were carried out using the Statistical Analysis System Version 9 [34]. Comparisons of variables were conducted using t-tests and Mantel-Haenszel chi-square. Generalized Estimating Equation (GEE) models were used to evaluate methemoglobin levels over the period of gestation. This accounted for the lack of normality assumption, correlation from repeated methemoglobin measurements on the same individuals, and imbalances due to missing data [35]. The contribution of other variables to methemoglobin variability was first explored with univariate regression by modeling individual predictor and exposure variables with baseline methemoglobin levels, and with the changes in methemoglobin over the period of gestation. The longitudinal analyses included only data collected from enrollment through delivery, and used the water source reported at each visit, not the water source reported at enrollment. Gestational age (in weeks) at enrollment and subsequent follow up visits was calculated using the date of last menstrual period reported at enrollment. The unstructured correlation matrix was used based on the assumption from the univariate assessment that correlation existed between each subject's methemoglobin levels, and the lack of equal spacing among the time points of observation. The least squared means option was used to obtain adjusted means that have been corrected for imbalances in the data.

Multi-variable regression analyses was used to evaluate the potential effects of nitrate intake from drinking water on methemoglobin levels among women who reported drinking tap water at home at the 36 weeks gestation follow up. Estimated nitrate ingested from water was calculated using the nitrate level in the water sample collected at 36 weeks, reported amount of water drank per day, divide by the body weight at 36 weeks follow up. Some variables such as age, water source, and drinking water contaminants that did not meet the 5% significance level were retained in the final models due etiological significance (i.e. there relationship to the outcome are of interest or they are potential confounders). Model goodness-of-fit was assessed for all final models.

## Results

### Study Population

Of the 577 eligible women who agreed to screening, 357 (62%) consented to participate. Characteristics of the study participants are presented in Table 1. Comparing private well users to public system users, differences were seen in age at enrollment (p = 0.03), weight at enrollment (p = 0.03), parity (p = 0.004), gravidity (p = 0.01), and alcohol use during pregnancy (p = 0.03). Overall 216 (60.5%) women reported never smoking tobacco, 92 (25.8%) smoked previously, and 49 (13.7%) were current smokers (smoked within the past month). Medical records review confirmed the usage of over-the-counter and/or prescription drugs in 270 (76%) women at some point during the current pregnancy. Of these, 233 (65%) used medications that could affect methemoglobin levels including those classified as "nitrosatable" drugs (i.e. a therapeutic drug that contains amides or amines). The majority used medications containing acetaminophen or antihistamines (n = 195 (84%)). Others included: antibiotics, anesthetics, sulfonamides, and beta-adrenergic-blocking [36].

**Table 1:** Participant Characteristics by Water Source Group

Characteristics	All n = 357	Private System n = 224 (63%)	Public System n = 133 (37%)	**p-value**^**a**^
Age at enrollment	29.4 ± 5.3	29.9 ± 5.4	28.6 ± 5.2	0.03

Age groups				0.04
	
<20	19 (5%)	11 (5%)	8 (6%)	
	
21-29	172 (48%)	99 (44%)	73 (55%)	
	
30-35	125 (35%)	86 (38%)	39 (29%)	
	
>35	41 (12%)	28 (13%)	13 (10%)	

Highest education group				0.67
	
<High School	15 (4%)	9 (4%)	6 (5%)	
	
≥High School	303 (85%)	196 (88%)	107 (80%)	
	
Refused/missing	39 (11%)	19 (8%)	20 (15%)	

Race				0.001
	
White	307 (86%)	203 (91%)	104 (78%)	
	
Other	11 (3%) 2 (1%) 9 (7%)			
	
Refused/missing	39 (11%) 19 (8%) 20 (15%)			

Ethnicity				0.03
	
Non-Hispanic	310 (87%)	203 (91%)	107 (80%)	
	
Hispanic	8 (2%)	2 (1%)	6 (5%)	
	
Refused/missing	39 (11%)	19 (8%)	20 (15%)	

Tobacco use at enrollment				0.47
	
Never	216 (60%)	133 (59%)	83 (62%)	
	
Previously	92 (26%)	58 (26%)	34 (26%)	
	
Present	49 (14%)	33 (15%)	16 (12%)	

Alcohol use during pregnancy				0.03
	
Yes	32 (9%)	18 (8%)	14 (11%)	
	
No	292 (82%)	192 (86%)	100 (75%)	
	
Missing/refused	33 (9%)	14 (6%)	19 (14%)	

Weeks gestation at enrollment				0.19
	
<8 weeks	41 (12%)	21 (9%)	20 (15%)	
	
8-10 weeks	57 (16%)	34 (15%)	23 (17%)	
	
11-13 weeks	259 (72%)	169 (76%)	90 (68%)	

Mean gestational age (weeks)	10.3 ± 1.6	10.4 ± 1.5	10.0 ± 1.6	0.06

Parity				0.004
	
0	92 (26%)	48 (22%)	44 (33%)	
	
≥1	226 (63%)	157 (70%)	69 (52%)	
	
Refused/missing	39 (11%)	19 (8%)	20 (15%)	

Vitamin use at enrollment				0.21
	
Yes	306 (86%)	196 (87%)	110 (83%)	
	
No	51 (14%)	28 (13%)	23 (17%)	

Gravidity				0.01
	
0	92 (26%)	48 (21%)	44 (33%)	
	
1	84 (23%)	56 (25%)	28 (21%)	
	
2	78 (22%)	61 (27%)	17 (13%)	
	
≥3	74 (21%)	47 (21%)	27 (20%)	
	
Refused/missing	29 (8%)	12 (5%)	17 (13%)	

Medication use ^b^				0.85
	
Yes	233 (65%)	147 (66%)	86 (65%)	
	
No	124 (35%)	77 (34%)	47 (35%)	

Weight at enrollment ^a,c,d^	165.7 ± 40.7	161.8 ± 37.4	172.6 ± 45.3	0.03

Weight gain during pregnancy ^a,c,d^	28.7 ± 11.8	28.8 ± 11.3	28.6 ± 12.4	0.76

^a ^p-value for comparison by water systems: t-test for continuous variables, Chi-square for categorical variables

^b^Include nitrosatable drugs and others that could affect methemoglobin levels

^c ^mean ± SD

^d^weight in pounds.

Eleven (3%) women experienced a miscarriage, 19 (5%) women were lost to follow-up, and of the 327 (91.6%) who completed the study through delivery, 27 (7.6%) delivered before 37 weeks gestation. The mean gestational age was 34 ± 2.5 weeks (range 27-36 weeks) for preterm deliveries, and 39 ± 1.3 weeks (range 37-42 weeks) for the other 300 women. There were 255 (71%) women who completed all 6 study interviews. At the 36 weeks gestation interview there were 105 women using public water systems, which is 21% less than at enrollment (n = 133). Compared to women using private wells at enrollment (n = 224), there were 11% less interviewed at the 36 weeks follow up (n = 199).

### Drinking Water Sources and Contaminants

At enrollment, 224 (63%) women reported using a private well and 133 (37%) reported a public water system as their water source. Twenty-eight women (8%) who completed the study reported a change in address during the study. At least 1 water sample was collected for 319 (89%) study subjects and 278 (78%) participants had 2 water samples. A positive correlation (Spearman's correlation rho = 0.82, p < 0.0001, n = 278) was observed between nitrate levels in the water samples collected at 36 weeks and postpartum. Water samples collected around 36 weeks gestation period had higher mean nitrate levels for samples collected in spring (1.69 ± 3.65), than summer (1.02 ± 3.01), fall (0.93 ± 1.76), and winter (0.96 ± 1.34) (p = 0.09).

Water use practices among women with data at enrollment and 36 weeks follow up are shown in Table 2. About 30% of participants reported using bottled water at home for drinking and cooking. Bottled water use away from home was common among participants, with more than 50% of women reporting use at enrollment and at 36 weeks follow up. A greater proportion of women on public water systems used bottled water away from home. An increase in use of water treatment devices was observed from enrollment to 36 weeks gestation but not in the use of devices that reduce nitrate levels (Table 2). A significantly greater proportion of private well users compared to public system users were using reverse osmosis or distillation (i.e., devices capable of removing nitrate from water) (p < 0.0001). At enrollment a greater proportion of women who reported using a water treatment device also reported drinking tap water (n = 154, 75%) compared to women who reported not using a treatment device (n = 86, 61%) (p = 0.007).

**Table 2:** Participants Water use Practices among Pregnant Women with data at Enrollment and 36 Weeks Gestation by Water Source Group (n = 304)

Characteristics	Enrollment			36 weeks follow-up		
	**Private Systems** **(n = 199)**	**Public System** **(n = 105)**	**p-value ^a^**	**Private systems** **(n = 199)**	**Public System** **(n = 105)**	**p-value**^**a**^

Water treatment			<0.0001			0.004

Yes	132 (66%)	48 (46%)		153 (77%)	64 (61%)	

No	67 (34%)	57 (54%)		46 (23%)	41 (39%)	

Treatment remove nitrate ^b^			<0.0001			0.02

Yes	36 (27%)	6 (13%)		36 (24%)	6 (9%)	

No	96 (73%)	42 (87%)		117 (76%)	58 (91%)	

Types of water treatment ^c^			<0.0001			0.006

Faucet filter	83 (31%)	36 (41%)		85 (29%)	39 (39%)	

Reverse osmosis or distillation	36 (13%)	6 (7%)		36 (12%)	6 (6%)	

Water softener	123 (46%)	44 (51%)		143 (49%)	56 (55%)	

Iron removal	22 (8%)	1 (1%)		23 (8%)		

Chemicals (chlorine)	4 (2%)			4 (2%)		

Water drink/cook at home			0.16			0.07

Tap water	136 (68%)	73 (70%)		138 (69%)	76 (72%)	

Bottled water	60 (30%)	32 (30%)		58 (29%)	29 (28%)	

Both	3 (2%)			3 (2%)		

Water drink away from home^a,d^			0.004			0.016

Tap water	61 (31%)	30 (29%)		62 (31%)	22 (21%)	

Bottled water	117 (59%)	72 (68%)		109 (55%)	75 (71%)	

Tap water from home	21 (10%)	3 (3%)		28 (14%)	8 (8%)	

Glasses of water/day Mean ± SD	5.5 ± 4.1	5.8 ± 4.0	0.58	6.7 ± 4.5	6.5 ± 4.3	0.79

^a^p-value comparing nitrate levels by water source (private vs. public) at enrollment and 36 weeks gestation

^b^Reverse osmosis or distillation

^c^Some participants have >1 treatment device

^d^Some participants drink water from >1 source

The distributions of nitrate and other contaminant levels are shown in Table 3. A significantly greater proportion of private well water tested positive for bacteria (p = 0.005), and 3 tested positive for fecal coliform. The private wells tested had a higher range of nitrate levels and 4 (2%) of the wells sampled had levels above the MCL. Water samples taken from homes with a water treatment device that removes nitrate had the lowest mean levels of nitrate (0.56 ± 0.97), compared to those using a treatment device that did not remove nitrate 1.02 ± 2.46, and those not using any treatment device (1.76 ± 3.3) (F = 3.66, p = 0.03).

**Table 3:** Distribution of Drinking Water Contaminants Tested by Water Source

Contaminant	Private Wells (n = 206)			Public Systems (n = 113)			
	
	Mean ± SD	Median	Range	Mean ± SD	Median	Range	*p*-value
Nitrate (mg/L)	0.9 ± 2.9	0.2	0.2-27.5	1.7 ± 1.6	1.1	0.2-5.2	0.001^a^

No treatment device	1.7 ± 4.3	0.3	0.2-20.4	1.8 ± 1.7	1.03	0.2-4.9	0.09^b^, 0.28^c^

Reverse osmosis or distillation	0.5 ± 1.0	0.2	0.2-6.1	0.7 ± 0.8	0.3	0.2-1.8	

Other Filter	0.7 ± 2.7	0.2	0.2-27.5	1.8 ± 1.6	1.2	0.2-5.2	

Copper (mg/L)	0.05 ± 0.2	0.005	0.001-2.2	0.1 ± 0.1	0.03	0.01-1.5	0.07^a^

Sulfate (mg/L)	136 ± 292	50.8	4-3570	141 ± 95	97.2	4-432	0.82^a^

Total Coliform Present (% positive)	28 (14%)			4 (3.5%)			0.005^a^

Fecal Coliform Present (% positive)	3 (1.5%)			0			

^a^p-value comparing mean contaminant levels by water source (private vs. public)

^b ^p-value comparing nitrate levels by treatment device used for private wells

^c^p-value comparing nitrate levels by treatment device used for public water systems

### Methemoglobin Levels in Pregnant Women

**Figure 1:**
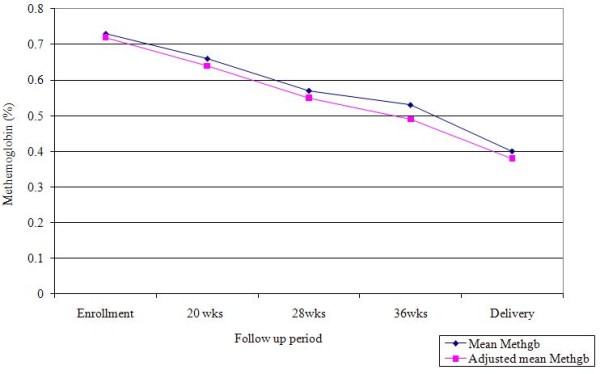
**Mean and adjusted mean % methemoglobin levels at each follow up visit for women with data at all time points (n = 255)**.

The average methemoglobin levels decreased with increasing gestational age (Table 4, Figure 1). This trend was still evident after mean methemoglobin levels were controlled or adjusted for covariates, β = -0.043, p = < 0.0001 (Table 5). At all follow up time points less than 1% of pregnant women tested had methemoglobin levels above the physiologic normal of 2% total hemoglobin (Table 4). Methemoglobin levels at 36 weeks gestation were higher among women with intermediate range of tap water nitrate levels compared to women with ≤3 mg/L nitrate, but no statistically significant difference was seen in the distribution of methemoglobin levels by nitrate subgroup (F = 2.56, 0.74) (Table 6). When the subgroup of 4 women with water nitrate level >10 mg/L were removed from the comparison, the difference in mean methemoglobin levels at 36 weeks between the lower and intermediate levels of nitrate subgroup was not statistically significant (t = 0.73, p = 0.47).

**Table 4:** Distribution of methemoglobin levels by follow up period

Follow up Period	N	Mean (SD)	Median	Range	n >2% methemoglobin
Time 1 (Enrollment)^a^	357	0.74 (0.48)	0.80	0.1-2.2	2

Time 2 (around 20 weeks)	317	0.67 (0.52)	0.60	0.1-3.6	3

Time 3 (around 28 weeks)	316	0.58 (0.46)	0.60	0.1-2.1	2

Time 4 (around 36 weeks)	304	0.51 (0.46)	0.50	0.1-2.2	2

Time 5 (day of delivery)	300	0.42 (0.47)	0.33	0.1-2.3	2

Time 6 (2-4 weeks postpartum)	295	0.39 (0.51)	0.28	0.1-3.0	5

^a^6-13 weeks gestation

**Table 5:** The Longitudinal analyses of methemoglobin and predictor variables (n = 327)

Parameter	β estimate	SE	95% CI	p-value
Intercept	-0.841	0.595	-2.007, 0.324	0.157

Gestational age (weeks)	-0.043	0.005	-0.054, -0.033	<0.0001

Carboxyhemoglobin	0.121	0.043	0.037, 0.205	0.005

Vegetable 24 hrs (yes)	-0.165	0.127	-0.414, 0.084	0.194

Cured meats (yes)	0.009	0.104	-0.196, 0.213	0.934

Water intake (cups/day)	-0.011	0.013	-0.037, 0.015	0.409

Drinking water (bottled)	-0.211	0.157	-0.519, 0.098	0.181

Water source (private well)	-0.099	0.134	-0.362, 0.162	0.455

Season methemoglobin tested (fall vs. winter)	0.388	0.143	0.107, 0.668	0.007

Season methemoglobin tested (spring vs. winter)	-0.146	0.153	-0.445, 0.154	0.340

Season methemoglobin tested (summer vs. winter)	0.039	0.154	-0.262, 0.342	0.796

Water treatment (none vs. nitrate removal)	0.029	0.205	-0.372, 0.430	0.887

Water treatment (other vs. nitrate removal)	0.321	0.185	-0.042, 0.684	0.082

Vitamin use (no)	-0.140	0.163	-0.459, 0.177	0.387

Nitrosatable drugs (no)	0.149	0.125	-0.096, 0.394	0.234

**Table 6:** Distribution of methemoglobin levels at 36 weeks follow up by tap water nitrate levels (n = 304)

	Methemoglobin (%)			
**Nitrate (mg/L)**	**Mean ± SD**	**Median**	**Range**	**p-value ^a^**

≤3 (n = 264)	0.46 ± 0.49	0.35	0.1-1.4	p = 0.74

>3≤ 10 (n = 38)	0.52 ± 0.46	0.50	0.1-2.2	

>10 (n = 4)	0.45 ± 0.33	0.50	0.1-0.8	

^a ^p-value comparing mean methemoglobin levels by nitrate subgroups

At enrollment, women who reported using a water treatment device that removes nitrate had the lowest mean levels of methemoglobin (0.61 ± 0.42); users of treatment devices that did not remove nitrate had the highest average levels of 0.81 ± 0.52; and women not using a treatment device had levels of 0.67 ± 0.45 (F = 4.12, p = 0.02). At enrollment, higher methemoglobin levels were observed in women who reported using tap water at home for drinking and cooking (0.77 ± 0.49), compared to women using bottled water (0.66 ± 0.47) (p = 0.04). However, at 36 weeks follow-up methemoglobin levels did not differ significantly between tap water users (0.51 ± 0.48) and bottled water users (0.46 ± 0.43) (p = 0.95).

Around 36 weeks gestation, nitrate intake from drinking water was estimated for women who reported drinking tap water at home (n = 214). The estimated nitrate intake from water ranged from 0.001 to 0.68 mg/Kg/day (mean = 0.02 ± 0.06, median = 0.01). Although the beta estimate from the multi-variable regression assessment of methemoglobin levels at 36 weeks was suggestive of higher methemoglobin levels among pregnant women with higher estimated nitrate intake from tap water, it was not statistically significant (β = 0.046, p = 0.986) (Table 7). Also, when the model was assessed with tap water nitrate levels as a continuous variable, there was no statistically significant association found with methemoglobin levels and nitrate levels at 36 weeks gestation among women who drank tap water at home (β = 0.787, p = 0.086).

**Table 7:** Multi-variable analyses of methemoglobin levels and covariates among women who reported drinking tap water at 36 weeks gestation (n = 214)

Parameter	β estimate	SE	p-value
Intercept	-1.512	0.439	0.001

Copper level (mg/L)	-0.299	1.172	0.799

Sulfate level (mg/L)	-0.0002	0.001	0.851

Age (years)	0.013	0.048	0.814

Race (White)	-0.858	1.608	0.601

Nitrate from water (mg/Kg/day)	0.046	2.564	0.986

Bacteria in water (yes)	0.361	0.526	0.493

Vegetable 24 hrs (yes)	-0.621	0.345	0.074

Water source (private well)	0.109	0.333	0.742

Carboxyhemoglobin	0.245	0.113	0.032

Cured meats (yes)	0.226	0.295	0.445

Vitamins (no)	-0.083	0.418	0.844

### Predictors of Change in Methemoglobin Levels

The longitudinal assessment showed decreasing methemoglobin levels with increasing gestational age. The model also showed methemoglobin levels of women tested in the fall were higher compared to those tested in the winter months (Table 5). Carboxyhemoglobin was included in the longitudinal model as an objective indicator of smoking status. Higher carboxyhemoglobin levels were predictive of higher methemoglobin levels. Longitudinal analyses of methemoglobin levels and the predicator variables of interest, including age, race, eating certain vegetables and cured meats in past 24 hours, water source, use of nitrosatable medication, use of tap vs. bottled water, and the use of water treatment devices were not statistically significant (Table 5). Although not statistical significant, the longitudinal results are suggestive of lower methemoglobin levels among women who reported vegetable intake within 24 hours of testing, and who consumed bottled water during pregnancy.

## Discussion

The study sought to add to the body of knowledge on methemoglobin levels and exposure to nitrates in drinking water during pregnancy by assessing methemoglobin levels (a biomarker for exposure to nitrates) in maternal blood over the period of pregnancy and evaluating the factors that could affect methemoglobin levels.

### Methemoglobin Levels in the Study Population

In order for methemoglobin levels to reach a level higher than what is considered the physiologic normal (2% of total hemoglobin) it has to be produced at a faster rate than the body is able to convert back to hemoglobin [12]. For nitrates to induce methemoglobinemia through exposure to drinking water, a high level has to be present in the drinking water to facilitate the rate of hemoglobin conversion to methemoglobin process. This was not demonstrated in the current study. Therefore the analytic and extrapolative power is limited by the relatively low nitrate levels found in water (only four participants had nitrate levels in their drinking water above the MCL), and the low levels of methemoglobin among participants.

Methemoglobin level in the blood is an accepted biomarker used in research for assessing exposure to nitrogen compounds or other substances that can oxidize hemoglobin. If exposure to an oxidizer such as nitrate is increasing methemoglobin level, it can be readily reduced back to hemoglobin once the oxidizer is removed. The transitory nature of methemoglobin and the multiple factors that can oxidize hemoglobin to increase methemoglobin levels makes the assessment of risk factors complex, especially when exposure levels are low and variable.

The current study result of decreasing methemoglobin levels during pregnancy did not support the *a priori *hypothesis that methemoglobin levels increase during pregnancy in populations exposed to nitrates in drinking water. Substantial variations in blood methemoglobin levels have been seen among the few studies that have measured methemoglobin levels during pregnancy. The lower methemoglobin levels reported in this study compared to values reported by other studies may be due to lower environmental and other exposures to nitrates in this study population, differences in the study population selection, in the exposure to substances that impact levels, and/or in the methods used to perform the actual testing [18,19,37,38].

The finding of decreased methemoglobin levels with increasing gestational age is not unique to this study. To evaluate the effects of sulfur dioxide and other air pollutants on methemoglobin levels during pregnancy, Mohorovic [37] measured methemoglobin levels 3 times during pregnancy at 1-month intervals in a group of women in Croatia. That study reported a decreased trend in methemoglobin concentrations among pregnant women during the period when sulfur dioxide levels were low which was classified as the "clean" air quality [37]. The low exposure is evident in the current study from the estimated nitrate intake from water among tap water drinkers (range 0.001 to 0.68 mg/Kg/day), which is significantly less than the RfD of 1.60 mg/Kg/day for nitrate [9].

Methemoglobin levels were not above the physiologic normal in the current study, and the results did not show a statistically significant association between higher methemoglobin levels in women with higher estimated nitrate intake from their tap water (Table 7). Tabacova et al. (1997) reported average methemoglobin values of 1.3% and levels as high as 6.6% in Bulgarian women with normal pregnancies and with pregnancy complications, respectively, but they were potentially exposed to drinking water nitrate levels from 8 mg/L-54 mg/L [18]. In another study, Gelperin et al. (1971) reported mean methemoglobin levels of 1.18% in mothers around the time of delivery in Danville, Illinois when the city water supply had nitrate levels above the MCL. Subsequent testing in other women around the time of delivery found a drop in average levels to 0.56% in mothers when the water system nitrate levels dropped below the MCL [39].

Methemoglobin levels that are not symptomatic may be indicative of exposure. It is not clear however what level of exposure to inducers such as nitrate will produce effects where there are no clinical symptoms, but there are adverse health effects especially over the long term [40]. Methemoglobin as a biomarker cannot address potential effects in pregnant women from the exposure to the lower than MCL nitrate levels in drinking water as observed in this study.

### Water Use Practices

Women on private water systems were more likely to use water treatment devices and used a device that removed nitrate. A previous study conducted in neighboring counties in this area of Minnesota reported similar findings with 22% of households on private wells using a water treatment device that removed nitrates [41]. In the current study, higher mean nitrate levels were found in samples taken from public water system users, but the range of nitrate levels was higher in private water systems (Table 3). The use of treatment devices that removes nitrates by private water system users is likely a contributing factor to the lower mean nitrate levels found in this subgroup. Residual chlorine levels were not tested in the current study, therefore the potential effects of chlorination on methemoglobin levels, especially as it relates to public water systems, cannot be effectively assessed. The potential effects of residual chlorine from the chlorination of public systems on the current study findings should be acknowledged.

The longitudinal analyses and the evaluation at 36 weeks gestation did not show an association between water source and methemoglobin levels when the effects of other factors including drinking water contaminants were evaluated (Tables 5 and 7). Previous studies examining drinking water contaminants and the association with a health outcome have usually relied on the approximate measure of the specific drinking water contaminant (e.g., nitrates in wells or public systems) as a proxy for exposure [2,42,43]. In this study, water source (private vs. public) did not emerge as a reliable significant predictor of methemoglobin levels which demonstrates the potential for exposure misclassification if only indirect methods of assessment are used. Data describing individual differences and variability in water consumption and practices are required to assess exposure and avoid misclassification.

The proportion of women who reported using in-home water treatment devices were similar to results from other published reports for private well users [44,45], but higher than reports for public system users [46,47]. Of interest is the increase in the proportion of women who reported using a treatment device later in pregnancy compared to baseline. It is possible that women started using a treatment device because they were pregnant and were likely more cautious about their water quality. Potential Hawthorne effect also cannot be ruled out [48]. That is, it is possible that women changed their habits as a result of being in this study. The number of women using treatment devices that remove nitrates did not increase so potential exposure to nitrates from drinking water was not likely affected. However, potential exposure to other methemoglobin inducers such as copper and chlorine that can be present in drinking water could have been affected by the increase use of other treatment devices.

The use of bottled water for drinking and cooking at home in the current study was higher than previously reported by Zender et. al. [46], but similar to that reported by other studies [49,50]. Also, bottled water use at home among private system users were similar to that reported elsewhere [44]. In the longitudinal assessment the type of water used (bottled vs. tap) for drinking and cooking was not statistically significant. However, the prevalence of bottled water use cannot be discounted as a contributing factor to the low levels of methemoglobin observed in this study.

### Nitrates and Other Water Contaminants

The nitrate levels and the proportion (2%) of samples with levels above the MCL found in the water taken from private wells were lower than previous surveys of private wells in more rural areas of the U.S., including the state of Minnesota [3,41,51,52]. An earlier survey reported 5.8% of private wells sampled in Minnesota exceeded the MCL for nitrate in drinking water [3]. The Nitrate Exposure and Infant Risk (NEXIR) survey reported 23% of wells sampled in two counties neighboring the area of this current study as having nitrate levels above the MCL, compared to 6% of wells sampled in more Suburban counties [41]. The rural area sampled may be comparable to this current study, but the discrepancy in findings may be partly due to the NEXIR report being based on samples taken directly from the wellhead and not from the inside tap. The NEXIR study also reported 22% of households in the more rural counties using in-home treatment devices that removed nitrates. It is plausible that samples taken from the inside tap would show a lower proportion exceeding the MCL.

This study supports previous findings that nitrate levels above the MCL and bacteria are more likely to be found in private water systems compared to municipal systems [8,52]. Squillace et al. [8] reported 11% of private well samples compared to 2% public wells exceeding the nitrate MCL. Also a report using data from the National Pesticide Survey reported 2.4% of private wells compared to 1.2% of community wells exceeded the MCL for nitrates [52]. A noteworthy finding in the current study is the majority of the water samples for the more intermediate levels of nitrate (3-10 mg/L) were from public water systems.

### Methemoglobin Levels in Relationship to Other Factors

Although more than 80% of participants reported frequent use of vitamins at enrollment and during pregnancy (Table 1), vitamin use did not show a significant effect on methemoglobin levels (Tables 5 and 7). However, previous reports have indicated that vitamins C and E can reduce methemoglobin levels [53,54], and both are commonly found in multivitamin preparations taken during pregnancy. It is possible that the extensive use of vitamins by the study group could partly explain the low levels of methemoglobin observed in the study population.

Dietary intake of some vegetables and cured meats is also an important source of nitrate exposure [55-57]. The results of the current study did not indicate higher methemoglobin levels in women who reported eating cured meats or vegetables that are potentially high in nitrates in the 24 hours before testing (Tables 5 and 7). This does not support the findings that consuming vegetables which are high in nitrates could impact methemoglobin levels [55,57-60]. The nitrate content in vegetables varies widely even in different areas of the same country [61], due to soil composition, farming practices, and the use of nitrogenous fertilizers. The source of food was not verified so no conclusions can be made regarding these findings and the areas soil nitrate level. The potential effects of vitamin rich vegetables on reducing methemoglobin level or on a woman's ability to reduce methemoglobin back to hemoglobin, is a factor also to be considered given the low levels of methemoglobin observed. Although this was not measured in the study, it could be a factor in complex physiologic process of methemoglobin formation.

## Summary

In this study the percent blood methemoglobin levels declined among the women as their pregnancy progressed. Drinking water contaminants such as nitrates and bacteria were more likely to be found at levels above the MCL in private drinking water systems. Water use practices (such as the use as in-home treatment devices) varied according to the source of the water for household use. The use of water treatment devices potentially affected exposure, and should be taken into account as a part of exposure assessment. Potential exposure to environmental nitrates via drinking water was low in the current study. At the nitrate levels documented in this study, there was no evidence of increased methemoglobin levels above what is considered physiologically normal.

Although this was a prospective study, nitrate and other drinking water contaminants were not measured longitudinally, therefore the potential effects of exposure to these contaminants could not be evaluated for the entire period of the pregnancy. Slight variation in the season of nitrate measurement was observed, with higher mean levels observed in water samples collected during the spring season, however, no statistical differences in mean levels were observed when compared to samples collected in winter, fall, or summer. The season of methemoglobin testing was included to the longitudinal assessment, but the results did not parallel the seasonal variation in nitrate levels, as methemoglobin levels were higher in fall and lower in spring and summer compared to winter (Table 5).

Differences in exposure to nitrates in food, medications, or other non-nitrogen containing hemoglobin oxidizers are not distinguishable based on methemoglobin levels [62,63]. The potential exposure to these factors known to increase methemoglobin formation were documented and evaluated in the study, and little variation existed in the study population for these factors.

## Conclusion

The results of this study were multi-faceted and of possible public health significance. A major strength of this study was its prospective longitudinal design, and the assessment of a range of factors that accompany the state of pregnancy. These included both behavioral factors and clinical factors, in addition to exposure to environmental factors. There is not a well established quantitative exposure response relationship for nitrate exposure via drinking water and the consequential development of methemoglobinemia [64].

Although avoidance or exposure lowering behavior was common (i.e., use of treatment devices and bottled water) which makes the estimation of risk more challenging, the lack of high levels of nitrates in the drinking water of the study population is the likely explanation for the lower methemoglobin levels observed. Our findings reiterate that identifying water source and asking participants if they drink their tap water is not enough to determine exposure to drinking water contaminants. Although the current study found nitrate levels above the MCL in private water system samples, higher mean levels of nitrates were found in samples taken from public water systems.

## Abbreviations

CDC: Centers for Disease Control and Prevention; U.S. EPA: U.S. Environmental Protection Agency; MCL: Maximum contaminant level; MDH: Minnesota Department of Health; NO_3_-N: Nitrate-nitrogen; SDWA: Safe Drinking Water Act; USGS: U.S. Geological Survey

## Competing interests

The authors declare that they have no competing interests.

## Authors' contributions

DMM, LCB, and RM conceived of and design the study. DMM supervised data collection, data management, statistical analysis, and led writing of the paper. CPM contributed to statistical analyses. LCB, LEF, RM and BL contributed to the writing of the paper. All authors reviewed and approved the final version.
